# Bacteria in the injection water differently impacts the bacterial communities of production wells in high-temperature petroleum reservoirs

**DOI:** 10.3389/fmicb.2015.00505

**Published:** 2015-05-21

**Authors:** Hongyan Ren, Shunzi Xiong, Guangjun Gao, Yongting Song, Gongze Cao, Liping Zhao, Xiaojun Zhang

**Affiliations:** ^1^State Key Laboratory of Microbial Metabolism, Joint International Research Laboratory of Metabolic and Developmental Sciences, School of Life Science and Biotechnology, Shanghai Jiao Tong UniversityShanghai, China; ^2^Institute of Petroleum Engineering and Technology, Shengli Oil Field Ltd.Sinopec, Dongying, China

**Keywords:** water flooding, petroleum reservoir, bacterial community, pyrosequencing, oil-bearing strata

## Abstract

Water flooding is widely used for oil recovery. However, how the introduction of bacteria via water flooding affects the subsurface ecosystem remains unknown. In the present study, the distinct bacterial communities of an injection well and six adjacent production wells were revealed using denaturing gradient gel electrophoresis (DGGE) and pyrosequencing. All sequences of the variable region 3 of the 16S rRNA gene retrieved from pyrosequencing were divided into 543 operational taxonomic units (OTUs) based on 97% similarity. Approximately 13.5% of the total sequences could not be assigned to any recognized phylum. The Unifrac distance analysis showed significant differences in the bacterial community structures between the production well and injection water samples. However, highly similar bacterial structures were shown for samples obtained from the same oil-bearing strata. More than 69% of the OTUs detected in the injection water sample were absent or detected in low abundance in the production wells. However, the abundance of two OTUs reached as high as 17.5 and 26.9% in two samples of production water, although the OTUs greatly varied among all samples. Combined with the differentiated water flow rate measured through ion tracing, we speculated that the transportation of injected bacteria was impacted through the varied permeability from the injection well to each of the production wells. Whether the injected bacteria predominate the production well bacterial community might depend both on the permeability of the strata and the reservoir conditions.

## Introduction

Microorganisms play various roles in petroleum reservoirs during oil exploration and post-operation processes (Magot et al., 2000). Recently, the microorganisms in petroleum reservoirs have received much attention, reflecting the effects of these microbes on oil recovery (Sen, 2008; Brown, 2010). Since the 1950's, water flooding has been a widely accepted method for increasing oil recovery from petroleum reservoirs. Flood water, obtained from the sea, river or groundwater, and recycled production water contain not only nutrients, dissolved oxygen and inorganic ions but also microorganisms (Grassia et al., 1996). These microorganisms are continuously injected into the subsurface and likely affect the reservoir ecosystem (White, 1984; Liu et al., 2005). Struchtemeyer et al. (2011) indicated that the addition of a mud component might shift the bacterial community structure in the reservoir during drilling. However, to date, little is known about the effects of the microorganisms introduced into petroleum reservoirs during water flooding (Struchtemeyer et al., 2011). Benefitting from the development of molecular techniques, such as denaturing gradient gel electrophoresis (DGGE) (Yoshida et al., 2005; Wang et al., 2008), clone libraries (Li et al., 2006, 2007b; Pham et al., 2009) and sequencing technology, an increasing number of studies have addressed the microbial composition of oil reservoir ecosystems (Li et al., 2007a; Pham et al., 2009; Kotlar et al., 2011; Kryachko et al., 2012; Wang et al., 2012; Gao et al., 2013; Lenchi et al., 2013; Lewin et al., 2014). Most of these studies have been on the microbial communities of production well samples. Recently, Lewin et al. indicated that the microbial composition and relative abundance in two non-linked production wells from a same geographical area were extremely similar (Lewin et al., 2014). Lenchi et al. reported that there were no significant differences in the bacterial composition between flooded and non-flooded production wells (Lenchi et al., 2013). However, in a previous study, using PCR-DGGE and clone library technology, the comparison of community structures of one injection water and two production water samples collected from a long-flooded petroleum reservoir indicated that each production well had a specific bacterial structure, despite both wells being continuously flooded with the same injection water for over 30 years (Ren et al.). We also detected significant differences in the bacterial composition between injection and production water samples (Ren et al.). Subsequently, Tang et al. (2012) confirmed this conclusion using clone library technology to compare two production wells in a block and three production wells in another block. These authors observed significant differences among the samples from different wells. In contrast to previous studies, these production wells were connected to an injection well (Tang et al., 2012). Considering the contradictions in previous studies, whether and how water flooding affects the bacterial community structures of individual production wells in the same block remains unknown. Nevertheless, understanding the effects of injected microorganisms on the microbial communities of production wells and revealing the impact factors associated with these effects are crucial to the practice of Microbial Enhanced Oil Recovery.

The aim of the present study was to corroborate the influence of the injected microbes on the microbial community structure of the subsurface during water flooding and to identify the factors that impact the microbial community structures in production wells. To this end, we compared the bacterial structure of samples from one injection well and six adjacent production wells in the same working block of a long-term water-flooded thermophilic oil reservoir. In addition, we attempted to associate the microbial community structures with the various parameters of the sampled wells.

## Materials and methods

### Site description and sample collection

In 1961, the Shengli oil field was established in Shandong province in the Yellow River Delta of China. The sandy oil-bearing horizon in the oil field is approximately 1173~1230 m, with an in situ temperature of 69°C and a pressure of 12 MPa. The block of Zhong Ng3 in the Shengli oil field has undergone water flooding for about 35 years. The water content in the formation water of this petroleum reservoir was over 96%.

**Figure 1:**
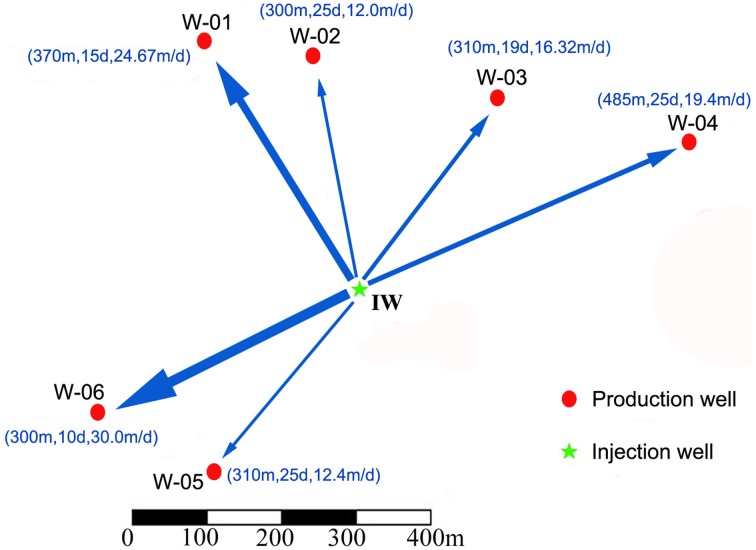
**Location maps and inter-correlations of the injection and production wells**. The arrow indicates the direction of water flow; the thickness of the arrow shows the rate of flow; and the numbers in the parentheses represent distance, tracer breakthrough time and water flow rates from the injection well to the production wells. IW, injection water sample; W-01 to W-06, production water samples.

The samples used in this study including one injection water sample (IW) and six production water samples (PWs) were collected from Zhong Ng3 working block. IW was collected from injection well (W-00) corresponding to injection well 6-313, and PWs were collected from six spatially independent production wells (W-01, W-02, W-03, W-04, W-05, and W-06, corresponding to production well 5-414, 6-13, 7N11, 8N11, 4-11, and 3-411, respectively). The relative positions of the injection well and the six production wells are shown in Figure 1. A tracer test was used to investigate the inter-correlation of the injection well and each production well. The tracer, thiocyanate (SCN^−^) was injected at one well along with the injection water and detected at a producing well after some period of time. Injection water was collected from the water supply pipelines before injection, and production waters were collected directly from the well heads of production wells. The names of the samples collected from the above wells were the same as the name of their corresponding well. All of the samples were collected in May 2008. Each sample was full filled sterile 5-L plastic bottle to prevent the oxygen and stored at 4°C before pre-treatment.

### Sample pre-treatment and DNA extraction

The microbial biomass of each sample was concentrated by filtration using the Millipore vacuum/pressure pump (Millipore Corporation, Bedford, Mass.). Each 250-mL sample was mixed with 1/4 volume of saturated sodium chloride solution and incubated at 70°C for 2 min before filtration (Ren et al., 2011). A 0.22-μm filter (Millipore, USA) was used to collect microbial biomass following an 8-μm filter (Bandao, China). All filters of one sample were collected and stored in a sterilized 10-mL centrifuge tube at −20°C for DNA extraction. The genomic DNA was extracted from the filters using a bead-beating protocol as described previously (Zhang et al., 2007). The AxyPrep PCR cleanup kit was used to purify genomic DNA, which was stored at −20°C until 16S rRNA gene amplification.

### Denaturing gradient gel electrophoresis (DGGE) analysis

The bacterial V3 region of the 16S rRNA gene used for DGGE analysis was amplified using the primers described by Muyzer et al. (1993). The 25-μL reaction mixture contained 0.5 U Taq DNA polymerase, 2.5 μL of the corresponding 10x buffer, 2 μL of a 2.5 mM dNTP mixture (Promega, USA), 1.6 ng /μL of BSA, 6.25 pmol of each primer, and 10 ng of genomic DNA. PCR was performed on Thermal Cycler (Bio-Rad, USA) using a touchdown procedure described previously (Liu et al., 2006), and “Reconditioning PCR” was performed as described by Thompson (Thompson et al., 2002). The concentrations of the PCR products were determined using a DyNA Quant 200 fluorometer (Pharmacia, US) and were evaluated using 1.2% (wt/vol) agarose gel electrophoresis.

V3-PCR products were separated in 8% (wt/vol) denatured polyacrylamide gels by electrophoresis using a Dcode System (Bio-Rad, Hercules, CA): the linear denaturant gradient was 27–55% (100% denaturant corresponds to 7 M urea and 40% deionised formamide). Electrophoresis was performed at a constant voltage of 200 V and a temperature of 60°C for 240 min in 1× Tris-acetate-EDTA (TAE) buffer. A total of 200–250 ng of PCR products were loaded in each lane, and the DNA bands were stained using SYBR green I (Amresco, Solon, Ohio) and photographed using a UV gel documentation system (UVItec, Cambridge, UK). The UPGMA tree was constructed using Quantity One (Bio-Rad, Hercules, CA).

### Pyrosequencing of the 16S rRNA gene V3 region

The V3 region of the 16S rRNA gene was amplified using the primers P1 (5′-CCTACGGGAGGCAGCAG-3′) and P2 (5′-ATTACCGCGGCTGCT-3′). A unique DNA barcode of eight nucleotides was added to the 5′ end of each primer and used to distinguish PCR products from different samples (Zhang et al., 2010). The 25-μL reaction mixture and PCR conditions have been described previously (Zhang et al., 2010). After the amplicon length and concentration were estimated, an equimolar mixture of all seven amplicon products was purified using the Gel/PCR DNA Fragments Extraction Kit (Geneaid, UKAS). Pyrosequencing was performed using the FLX Titanium system (Roche) (Margulies et al., 2005).

All the raw sequences were checked with the standards below: (i) matching primer; (ii) less than one for the edit distance of proximal and distal barcode; (iii) containing at least 100 bases; (iv) having no more than two ambiguous bases. Subsequently, V3 sequences were extracted and sorted to different samples according to barcodes. Sequences with 100% identity were considered as a unique sequence, and then was associated with the number of times observed in each sample (Zhang et al., 2010). Unique sequences were aligned using alignment function of web service in Greengenes (http://greengenes.lbl.gov). Aligned sequences were uploaded to ARB for calculating the distance matrix. Operational taxonomic units (OTUs) were divided using DOTUR based on 97% sequence identity (Schloss and Handelsman, 2005). Rarefaction curves and the Shannon index were also generated using DOTUR to estimate the diversity and richness of each sample. One representative sequence was randomly selected from each OTU on ARB, and its nearest phylogenetic neighbors were searched against database of RDP (http://rdp.cme.msu.edu). The relationship between various samples was illustrated by Principal coordinate analysis (PCoA) using UniFrac distance.

### Nucleotide sequence accession number

The sequences obtained in this study have been submitted to the GenBank databases under accession number SRP006479.

## Results

### Characteristics of the injection well and production wells

The location of the injection well and six production wells is shown in Figure 1. The distances from the injection well to the six production wells were between 300 and 480 m. According to the ion tracing results, the tracer breakthrough times were between 10 and 25 days, indicating that the water flow rates from the injection well to the six production wells ranged from 12 to 30 m/d (meters per day) (Figure 1). The working block contained three oil-bearing strata in the subsurface. W-01 and W-04 shared the same oil-bearing strata as W-02 and W-05, whereas W-03 and W-06 had distinct oil-bearing strata (Table 1). The physicochemical characteristics of these samples are also shown in Table 1.

**Table 1:** **Characteristics of sampling injection and production wells**.

	**IW**	**W-01**	**W-02**	**W-03**	**W-04**	**W-05**	**W-06**
**CHEMICAL SPECIES AND CONTENT (mg/L)**							
Cl^−^	4149.5	3933	3818	3683	3787	3578	3936
HCO^−^_3_	1073	988	931	1025	1061	1129	1042
Ca^2+^	104.5	68	72	128	68	76	128
Mg^2+^	36	55	41	17	43	17	19
K^+^+Na^+^	2921	2790	2728	2624	2719	2627	2762
SO^2−^_4_	3.5	106	125	58	48	0	0
TDS^*^	8301	7938	7717	7535	7725	7525	7888
Oil bearing		Ng33		Ng33	Ng33		
		Ng34	Ng34		Ng34	Ng34	
strata#		Ng35	Ng35	Ng35	Ng35	Ng35	Ng35

* *total dissolved solids*;

# *the strata of well sampling from*.

### PCR-DGGE analysis of the injection and production water samples

**Figure 2:**
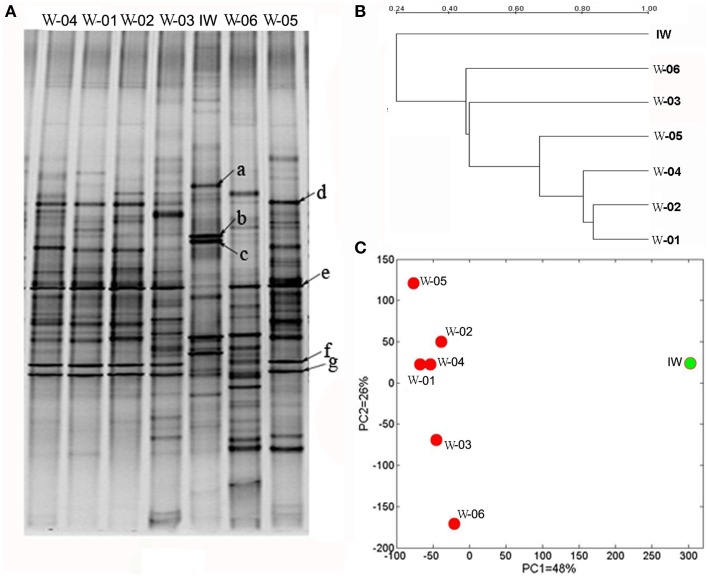
**DGGE profiles of PCR-amplified 16S rRNA gene fragments of bacterial communities from one injection water sample and six production water samples. (A)** DGGE profiles of the bacterial communities, **(B)** clustering analysis of the DGGE digitalized data, and **(C)** scatter plot of the results from the principal component analysis (PCA) of the DGGE digitalized data. IW, injection water sample; W-01 to W-06, production water samples.

PCR-DGGE is a useful tool for comparing the microbial structures of different samples. As shown in Figure 2A, the PCR-DGGE profiles for the 16S rRNA gene V3 region revealed distinct bacterial structures between the IW and PW samples. The three predominant bands (a, b, and c) in the IW sample were weaker or absent in the PW samples. However, the four predominant bands (d, e, f, and g) in the PW samples were weaker or absent in the IW sample. The principal component analysis (PCA) of the DGGE fingerprints clearly differentiated the IW and PW samples (Figure 2B). Principal components (PCs) 1 and 2 accounted for 48 and 26% of the total variance, respectively. The bacterial structure of W-01 and W-04 was more similar among all samples in the PW samples. The clustering analysis of DGGE profile showed that the similarity between the IW and the PW samples ranged from 37 to 46%, which was lower than the 54 to 75% similarity among PW samples (Figure 2C). The results from the *t*-test of the distances among the wells showed a significant difference between the two types of wells.

### Bacterial structures in the injection and production water samples

The sequences of the V3 fragment of the 16S rRNA gene in the IW and PW samples were obtained using barcode pyrosequencing. A total of 5753 useable reads were obtained, with 1836 unique sequences. A total of 543 operational taxonomic units (OTUs) were defined based on 97% identity (Figure S1A). The coverage of each library was higher than 85%. The richness of the bacterial communities in each sample was estimated using a rarefaction analysis (Figure S1B). The rarefaction curves did not approach a plateau, despite the high number of reads. The curves of the Shannon diversity index of all samples reached saturation. No significant differences in the Shannon diversity index were detected among all samples, except for W-03, which showed slightly higher diversity than the other samples. These results suggest a higher complexity within the bacterial community in W-03 (Figure S1). The detailed taxonomic information for these OTUs is shown in Table S1.

**Figure 3:**
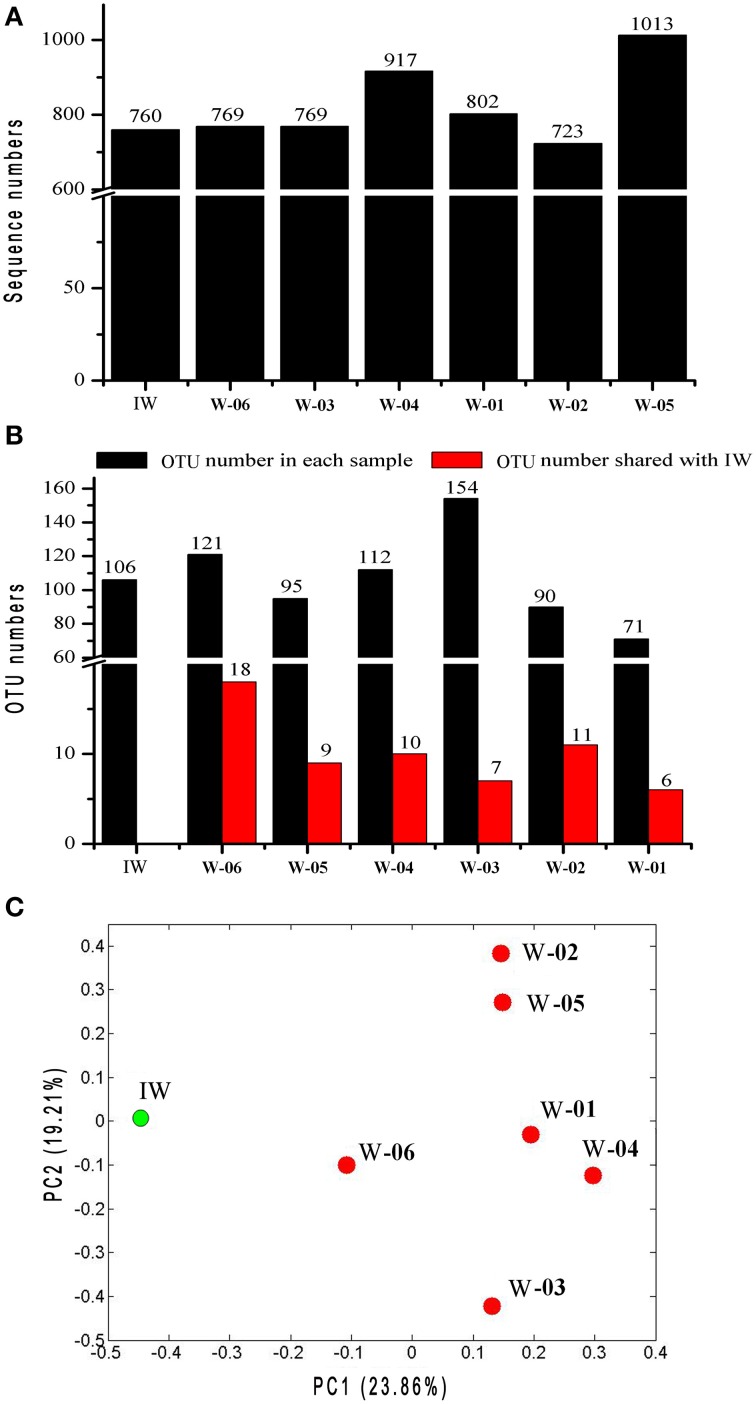
**Comparison of microbiota between the injection and production water samples based on pyrosequencing data and UniFrac metrics. (A)** Number of sequences in each sample. **(B)** OTUs in each sample and OTUs shared between the injection water sample and each production water sample. **(C)** The PCoA plot was generated using weighted UniFrac analysis. IW, injection water sample; W-01 to W-06, production water samples.

The unweighted UniFrac principal coordinates analysis (PCoA) showed that the bacterial community in the IW sample was different from that in the PW samples (Figure 3). UniFrac significance tests for Unifrac distances indicated that the bacterial community of the IW sample was significantly different from that of each PW sample (*P* < 0.05). However, the bacterial communities of W-01 and W-04 showed no significant differences. This result is similar to the comparative findings of the bacterial communities of W-02 and W-05 (*P* ≥ 0.05).

**Figure 4:**
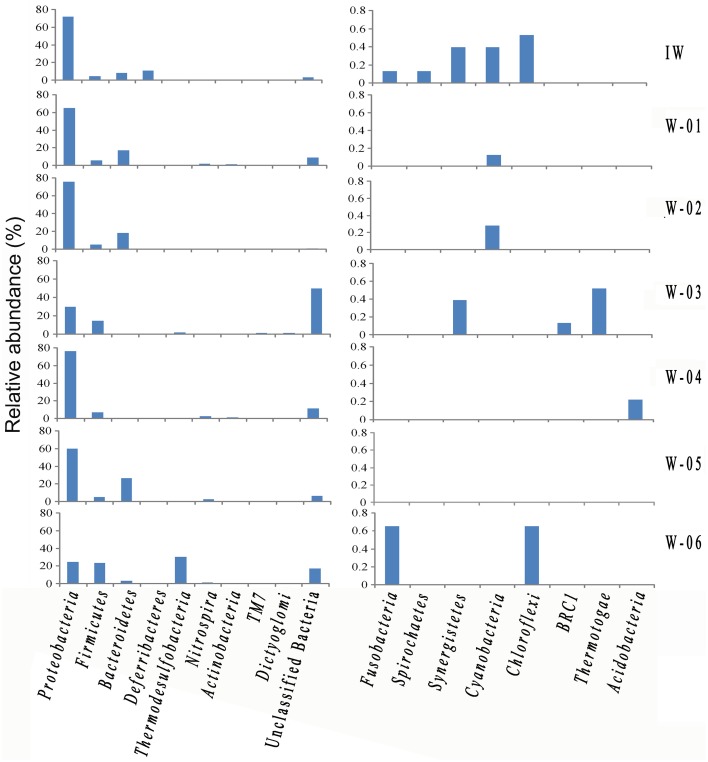
**The identity and abundance of representative phyla in the injection and production water samples**. IW, injection water sample; W-01 to W-06, production water samples.

**Figure 5:**
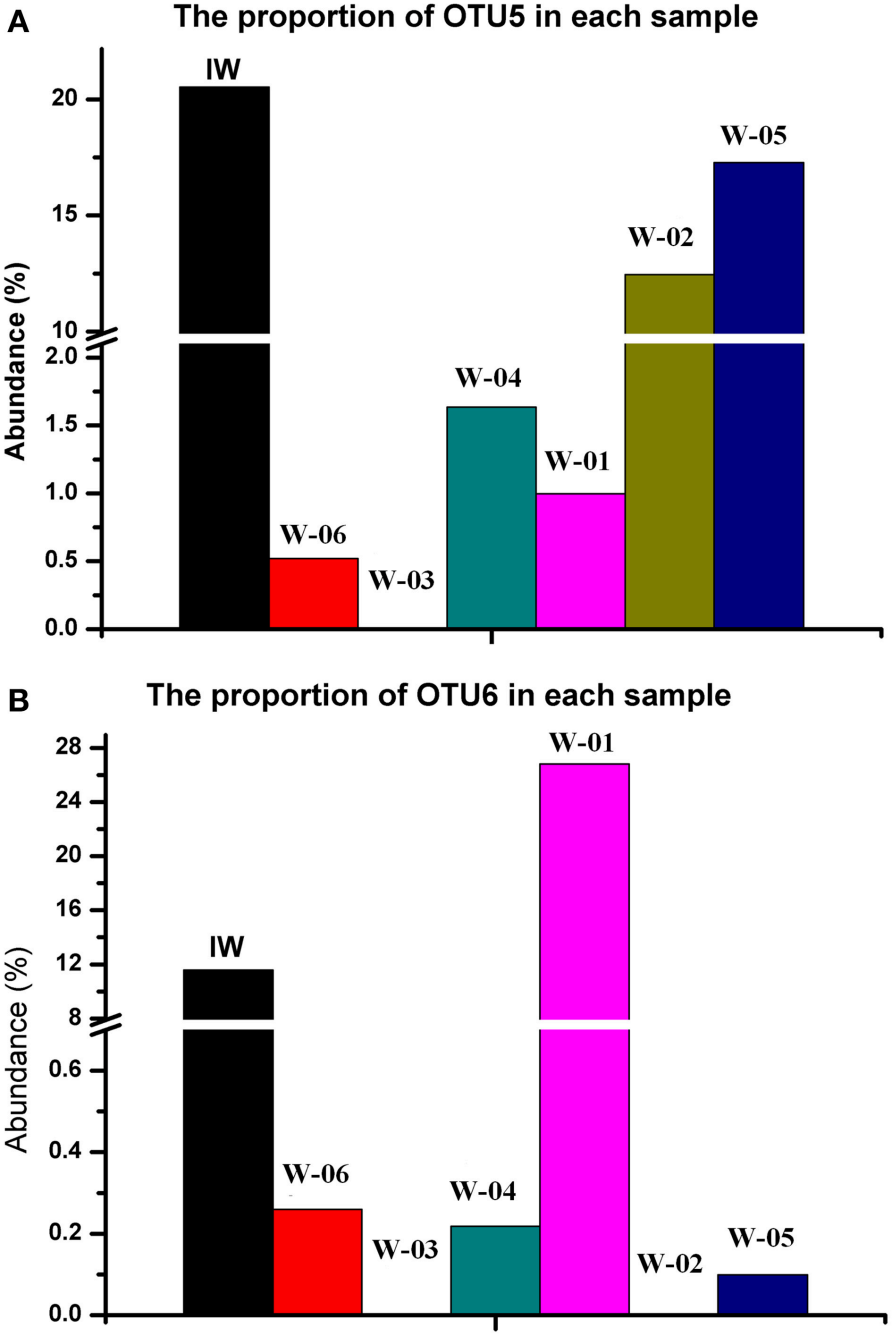
**Comparison of abundances of two OTUs from** ***Pseudomonas*** **in the injection and production water samples. (A)** OTU5; **(B)** OTU6. IW, injection water sample; W-01 to W-06, production water samples.

A total of 776 (13.5%) sequences shared less than 75% identity with the nearest reference and could not be assigned to any known phylum. The remaining sequences represented 17 phyla, of which Proteobacteria and Firmicutes were detected in injection and production water samples, the remaining 15 phyla showed a remarkable transition from injection to production water samples (Figure 4). On the genus level, 40.4% of the sequences were classified into 59 genera (Table S1). Among these genera, *Pseudomonas* was the only predominant genus in the IW sample, whereas the predominant genera in the PW samples were *Pseudomonas, Acinetobacter, Halomonas, Thermodesulforhabdus, Thermacetogenium, Thermodesulfovibrio, Chryseobacterium* and *Thermodesulfobacterium*. Particularly, production wells W-01 and W-04 shared the same oil-bearing strata harboring the common genus, *Halomonas*, with the proportions of 18.5 and 4.7%, respectively. Similar results were also observed for production wells W-02 and W-05, which shared the common genus *Chryseobacterium*, with proportions of 18.3 and 26.2%, respectively. A total of 106 OTUs were detected in the IW sample, of which only 5.7 to 17.0% were detected in the PW samples, suggesting that most of the OTUs introduced were not observed in production wells. Among the OTUs detected in the IW sample and at least one PW sample, the abundance of the five OTUs, associated with Pseudomonadaceae, Alteromonadaceae, and Rhodocyclaceae, decreased in the PW samples compared with those in the IW sample (Figure S2A). Conversely, the abundance of 11 OTUs, associated with Pseudomonadaceae, Enterobacteriaceae, Hydrogenophilaceae, Moraxellaceae, Syntrophobacteraceae and Rhodocyclaceae, increased in the PW samples compared with those in the IW sample (Figure S2B). Among these OTUs, two OTUs (OTU5 and OTU6) associated with *Pseudomonas* accounted for 20.5 and 11.6%, respectively, of the total sequences in the IW sample. However, the abundance of OTU5 and OTU6 in the PW samples was significantly different. Particularly, OTU5 and OTU6 were weakly or undetectable in W-06, W-04, and W-03. However, either one or both of these OTUs showed high abundance in W-01, W-02, and W-05 (Figure 5). Significant differences in the abundance and ratio of these two OTUs between PW and IW samples suggested that these two bacteria had different fates in different production wells.

Several days passed between water injection and water recovery. To examine the changes in the microbial structure of the injection water over time, two injection water samples (IW/06 and IW/08) from the same injection well were collected at two different sampling times in December 2006 and May 2008, respectively, and the bacterial communities were analyzed using barcode pyrosequencing. A total of 991 and 760 sequences were compared to examine the community structure in these two samples (Figure S3A). The main bacteria phyla in the injection well were consistent at both sampling times, although there was a slight difference in terms of abundance (Figure S3B). One-way ANOVA based on the abundance of each OTU in the two samples from the same injection well indicated no significant difference between them, suggesting that the community structures in the water supply system barely changed after 18 months.

## Discussion

The water flooding of the Shengli petroleum reservoir has been continuous for more than 30 years. The production water was separated and subsequently recycled as injected water without sterilization. As bacteria proliferate within the pipeline and tanks of the water supply system, numerous microbial cells in the injection water are continuously introduced into the reservoir. However, the impact of this process has not been well characterized.

To compare the influence of injected bacteria on the microbial structure of production wells and associated reservoirs, we considered the stability of the bacterial community structure in injection water. Although it took 10 to 25 days for the injection water to arrive at each production well according to an ion tracer test, a relatively constant injection of bacterial communities during this long period of time, as shown in the present study, warranted the reliability of a comparative study on injection water and production water, even when sampled at the same time. The influence of the sampling time on the effect of the injection water samples on the bacterial structure of the production wells was negligible, as previously reported (Lysnes et al., 2009).

Previous phylogenetic analyses identified a large number of unclassified bacteria in the production wells of petroleum reservoirs (Dahle et al., 2008; Pham et al., 2009). In the present study, 5753 sequences were obtained from seven Shengli petroleum reservoir samples using pyrosequencing technology. Among the total sequences, 63.2% of the sequences in the IW sample and 59.0% of the sequences in the PW samples might belong to new genera, indicating that further analysis is necessary to understand the microbial ecology in petroleum reservoirs due to their high diversity.

The results obtained in the present study showed that Proteobacteria, Firmicutes, and Bacteroidetes were the predominant phyla in Shengli petroleum reservoirs, consistent with previous reports on high-temperature petroleum reservoirs (Li et al., 2006, 2007b; Dahle et al., 2008). In addition, although detected in low abundance (<1.0%), some phyla in the Shengli petroleum reservoir, such as Saccharibacteria (formerly named TM7) and BRC1, have recently been reported in other high temperature oil reservoirs (Tang et al., 2012; Wang et al., 2012; Lenchi et al., 2013). Presenting more detail at the genus level, the present study showed many more genera than previous reports. The predominant genera, including *Pseudomonas, Halomonas, Acinetobacter*, and *Desulfothiovibrio*, have been previously described in other petroleum reservoirs (Orphan et al., 2000, 2003). Additionally, many other taxa, such as *Enhydrobacter, Pelomonas*, and *Weissella*, have rarely been detected in similar environments, indicating the complexity of petroleum microbial structures, and the strong power of the pyrosequencing method for elucidating the microbial community.

The results of both PCR-DGGE and massive parallel pyrosequencing suggested that the bacterial communities in the IW and PW samples were different. Similar conclusions have been reported using fingerprinting (She et al., 2005; Yuan et al., 2007) and a clone library approach (Ren et al., 2011; Tang et al., 2012). However, using pyrosequencing, we provided more detailed taxonomic information in the present study. We observed that the abundance of 11 OTUs increased in the PW samples compared with the IW sample, indicating that some of the bacteria had adapted to the subsurface environment and might have proliferated there. However, 69.8% of the OTUs in the IW samples were different from those in the PW samples, indicating that many phylotypes of bacteria injected into the reservoir did not survive and were undetected in the production wells. In addition, the number of bacterial cells in the production water was at least one order of magnitude less than that in the injection water, although the reservoir had been continuously flooded for three decades, indicating a strong interception of microorganisms in the strata. Moreover, the two most predominant bacteria in the IW sample, namely OTU5 and OTU6, had different ratios in the PWs samples. OTU6 was almost undetectable in the W02 sample, whereas OTU5 was as high as 12% in the same sample. Considering the large difference in the number of bacterial cells between the injection and production water samples, we speculate that the bacterial biomasses of these two OTUs in the PW samples are not directly flushed from the injected biomass, further suggesting that only a small portion of the cells were transported to the production wells, even bacteria that were highly abundant in the injection water. Most of the cells likely were blocked during transit, reflecting the low permeability of the strata; therefore, only a few injected bacterial cells were transported to the far end in the production wells. The ion tracer test showed that the water flow rates from the injection well to the six production wells were different, suggesting the distinct porosity and water transferability of the formations along the pathway to the different production wells. Thus, in addition to their scarcity, the numbers of cells transported to these six production wells also varied. The high abundance of specific bacteria in the different production wells likely reflects the in situ reproduction of these microbes. The nanodarcy permeability and extremely small average pore throat size of the shale have prevented the pervasion of bacteria (Jack et al., 1991; Corinne Whitby, 2009; Brown, 2010). Consequently, the various bacterial communities formed within these wells, adjacent to the same injection well, undergo long-term water flooding. In the present study, the comparison of samples from multiple production wells and the different abundance of specific OTUs strongly supported this hypothesis. In the practice of Microbial Enhanced Oil Recovery (MEOR), selected bacteria are often injected into the oil reservoirs to enhance the recovery of crude oil. Based on the present study, we propose that the success of the injected bacteria in the oil reservoir depends on two factors: increased movement of the injected bacterial cells and proper environmental conditions for bacterial growth. Therefore, the previous unpredictable results of MEOR might reflect the low permeability of bacterial cells to the targeted reservoirs.

The results obtained in the present study also indicated similar bacterial structures in W-01 and W-04, which received water-oil fluid from the same oil-bearing strata. The same phenomenon occurred for W-02 and W-05 (Table 1). However, the other two production wells, W-03 and W-06, with distinct oil-bearing strata, contained unique bacterial communities. These results suggest that the bacteria in the production wells are closely associated with the oil-bearing strata that harbor them.

In conclusion, the differences in structure of the bacterial communities in the injection well and six associated production wells indicated that the bacterial composition in the production wells is strongly associated with the corresponding oil-bearing strata and the permeability from the injection well to the production wells. We emphasize that understanding bacteria cell flow mechanisms in situ might be key to the optimal design and evaluation of field applications of MEOR.

### Conflict of interest statement

The authors declare that the research was conducted in the absence of any commercial or financial relationships that could be construed as a potential conflict of interest.
