# Supplementary material for: Bacteria in the injection water differently impacts the bacterial communities of production wells in high-temperature petroleum reservoirs
Source: Front Microbiol. 2015 May 21;6:505. doi: 10.3389/fmicb.2015.00505 (PMC4439544; doi:10.3389/fmicb.2015.00505)
Supplement: Supplementary file 1 [file Presentation1.PDF]

# Using Pyrosequencing to Explore the Effect of Water Flooding on Microbial Communities of a High-Temperature Petroleum Reservoir

Hongyan Ren[1], Shunzi Xiong[1], Guangjun Gao[2], Yongting Song[2], Gongze Cao[2], Liping Zhao[1] , Xiaojun Zhang[1*]

*1, State key laboratory of Microbial metabolism and School of Life Science & Biotechnology, Shanghai Jiao Tong University, Shanghai 200240, China*;

*2, Institute of Petroleum Engineering and Technology, Shengli Oil Field Ltd., Sinopec, Dongying 257000, China*

* Corresponding authors:

Xiaojun Zhang,

Tel.:+86 21 34204878(O);

Fax number: +86 21 34204878;

E-mail address: xjzhang68@sjtu.edu.cn

Mailing address: 800 DongChuan Road, Shanghai Jiao Tong University,

Shanghai 200240, China

## Supplemental Materials:

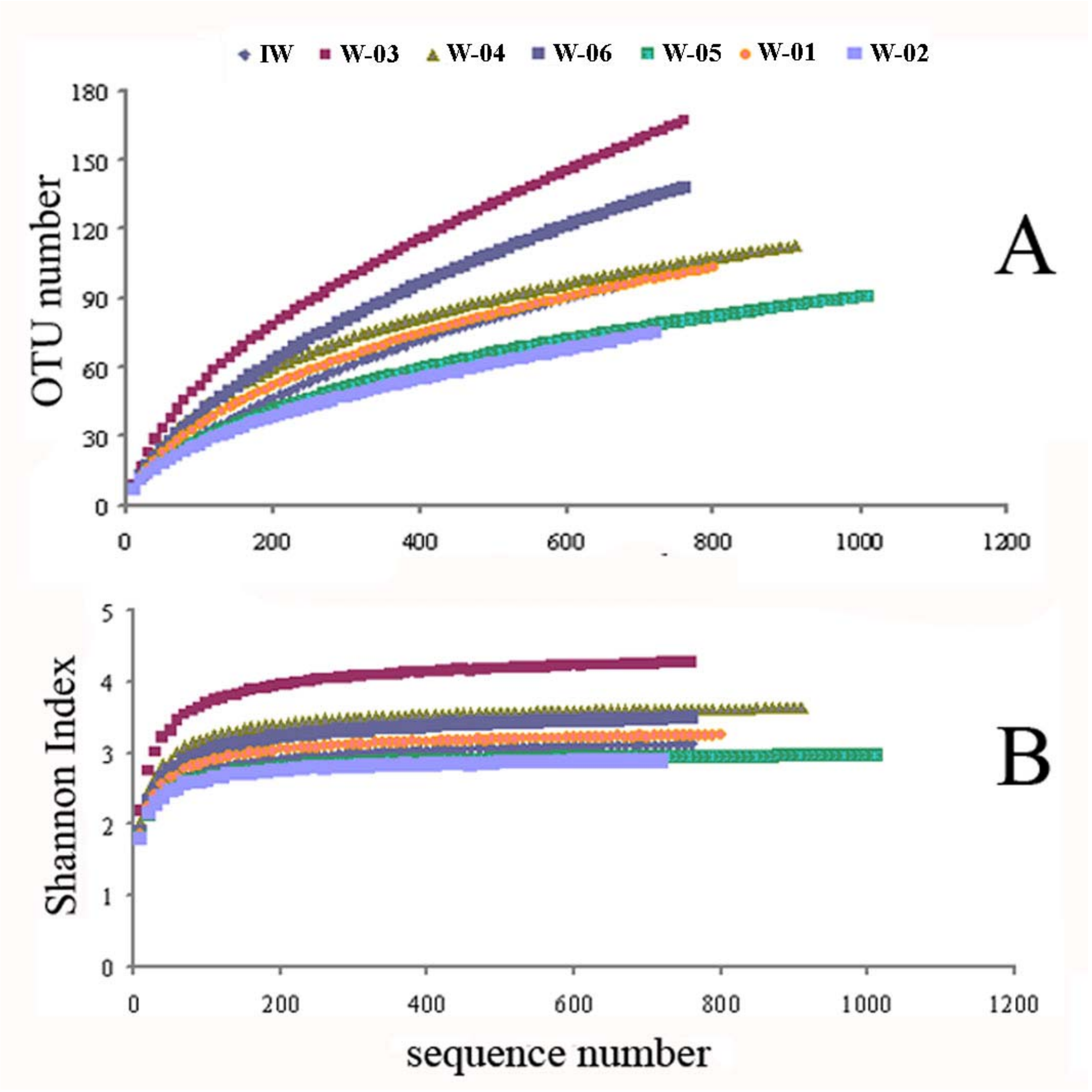


**Figure S1 Rarefaction (A) and diversity (B) Curve of injection water and oil well production water samples.**

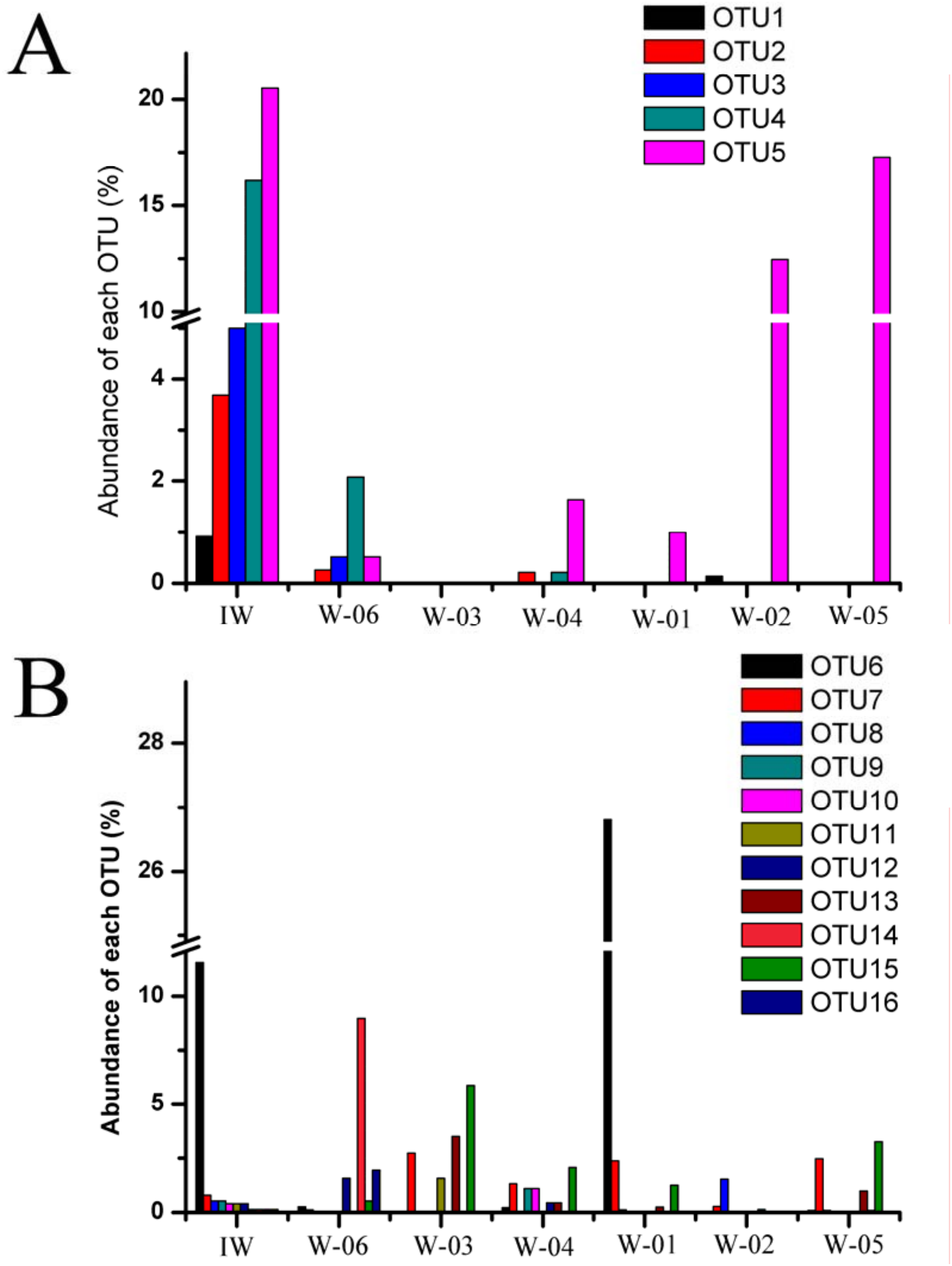


**Figure S2 The 16 most abundant OTUs in the samples. The abundance of the OTUs reduced in the production water samples (A), and increased in either one of production water samples (B).**

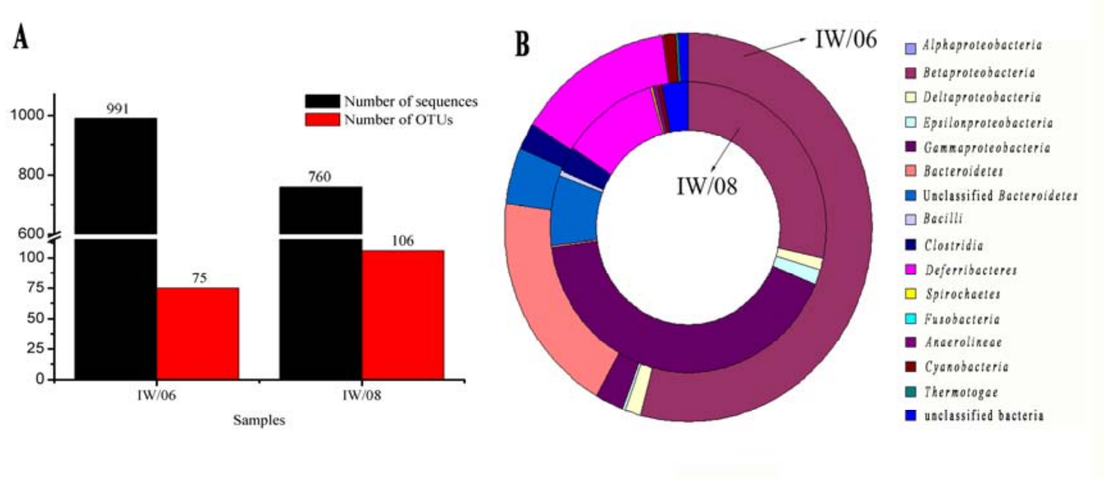


**Figure S3 Comparison of injection water samples sampled in 2006 and 2008. A, the number of 16S rRNA gene sequences and OTUs in two samples; B, bacterial community structures of two samples.**

# Table S1. Identified genera and their abundance in samples

| Phylum | Genera | Injection water (%) | Production water samples (%) | | | | | |
|---|---|---|---|---|---|---|---|---|
| | | IW | W-01 | W-02 | W-03 | W-04 | W-05 | W-06 |
| Thermotogae | *Thermotoga* | 0 | 0 | 0 | 0.5 | 0 | 0 | 0 |
| Thermodesulfobacteria | *Thermodesulfobacterium* | 0 | 0 | 0 | 0 | 0.1 | 0 | 30.0 |
| Synergistetes | *Anaerobaculum* | 0 | 0 | 0 | 0.1 | 0 | 0 | 0 |
| | *Thermovirga* | 0.3 | 0 | 0 | 0.1 | 0 | 0 | 0 |
| Proteobacteria | *Brevundimonas* | 0 | 2.2 | 0 | 0.1 | 2.0 | 1.1 | 0 |
| | *Hyphomonas* | 0 | 0 | 0 | 0 | 0 | 0 | 0.1 |
| | *Bosea* | 0 | 0 | 0.8 | 0 | 0.4 | 0.1 | 0 |
| | *Bradyrhizobium* | 0 | 0.1 | 0.5 | 0 | 1.0 | 0.3 | 0 |
| | *Paracoccus* | 0 | 0.1 | 0 | 0 | 1.2 | 0 | 0 |
| | *Roseomonas* | 0 | 0 | 0 | 0.1 | 0 | 0 | 0 |
| | *Oceanibaculum* | 0 | 0.2 | 0 | 0 | 0.4 | 0 | 0 |
| | *Sphingomonas* | 0 | 0.4 | 0.8 | 0 | 0 | 1.6 | 0.1 |
| | *Achromobacter* | 0 | 0 | 1.4 | 0 | 0 | 0.7 | 0 |
| | *Ralstonia* | 0 | 0 | 0.5 | 0 | 0 | 0.1 | 0 |
| | *Pelomonas* | 0 | 0 | 0.7 | 0.3 | 0 | 0.4 | 0 |
| | *Hydrogenophilus* | 1.1 | 0 | 0 | 0 | 0 | 0 | 0 |
| | *Azospira* | 0 | 0 | 2.2 | 0 | 0.3 | 1.0 | 0.1 |
| | *Zoogloea* | 0.1 | 0 | 0 | 0 | 0 | 0.1 | 0 |
| | *Desulfomicrobium* | 0.7 | 0 | 0 | 0 | 0 | 0.5 | 0.8 |
| | *Desulfovibrio* | 0 | 0 | 0 | 0 | 0 | 0 | 0.4 |
| | *Desulfacinum* | 0 | 0 | 0 | 0 | 0 | 0 | 0.1 |
| | *Thermodesulforhabdus* | 0.1 | 0 | 0 | 0 | 0 | 0.1 | 9.6 |
| | *Arcobacter* | 0.8 | 0 | 0 | 0 | 0 | 0 | 0.1 |
| | *Marinobacter* | 0 | 0.5 | 0 | 0 | 0.1 | 0 | 0.3 |
| | *Marinobacterium* | 0 | 0 | 0 | 0 | 0 | 0 | 0.1 |
| | *Thiofaba* | 0.5 | 0 | 0 | 0 | 0 | 0 | 0 |
| | *Halomonas* | 0 | 18.5 | 0 | 0 | 4.7 | 0 | 0 |
| | *Acinetobacter* | 0.1 | 0.7 | 0.1 | 4.4 | 1.4 | 1.6 | 0 |
| | *Enhydrobacter* | 0 | 0 | 0 | 0 | 1.4 | 0 | 0 |
| | *Pseudomonas* | 32.4 | 27.9 | 13.8 | 0 | 1.9 | 17.4 | 0.8 |
| | *Stenotrophomonas* | 0 | 0 | 0.5 | 0 | 0 | 0.5 | 0 |
| Nitrospira | *Thermodesulfovibrio* | 0 | 2.1 | 0 | 0.1 | 23 | 2.5 | 1.2 |
| Firmicutes | *Geobacillus* | 0 | 0.2 | 0 | 0 | 0 | 0 | 0 |
| | *Brevibacillus* | 0 | 0 | 0 | 0 | 2 | 0 | 0 |
| | *Staphylococcus* | 0 | 0.4 | 0.3 | 0.4 | 21 | 0.3 | 0 |
| | *Aerococcus* | 0 | 0.2 | 0 | 0 | 0 | 0 | 0 |
| | *Atopostipes* | 0 | 0 | 0 | 0 | 0.1 | 0 | 0 |
| | *Leuconostoc* | 0 | 0 | 0 | 0 | 0.4 | 0 | 0 |
| | *Weissella* | 0.3 | 0.1 | 0 | 0 | 0 | 0.7 | 0 |
| | *Lactococcus* | 0 | 5 | 0 | 0.1 | 0 | 0.4 | 0 |
| | *Streptococcus* | 0 | 0 | 0.7 | 0 | 13 | 0 | 0 |
| | *Clostridium* | 0.1 | 0 | 0 | 0 | 0 | 0 | 0 |
| | *Alkalibacter* | 0.3 | 0 | 0 | 0 | 0 | 0 | 0 |
| | *Soehngenia* | 0 | 0.2 | 0 | 0 | 0 | 0 | 0.3 |
| | *Faecalibacterium* | 0 | 0 | 0.1 | 0 | 0 | 0.2 | 0 |
| | *Syntrophothermus* | 0 | 0 | 0 | 1.4 | 0 | 0 | 0 |
| | *Megamonas* | 0 | 0 | 0 | 0 | 0 | 0 | 0.1 |
| | *Veillonella* | 0 | 0.1 | 0 | 0 | 0 | 0 | 0 |
| | *Caldanaerobacter* | 0 | 0 | 0 | 0.3 | 0 | 0 | 1.6 |
| | *Thermacetogenium* | 0 | 0 | 0 | 0 | 0 | 0 | 7.8 |
| | *Coprothermobacter* | 0 | 0 | 0 | 0 | 0 | 0 | 5.6 |
| Dictyoglomi | *Dictyoglomus* | 0 | 0 | 0 | 0.5 | 0 | 0.1 | 0 |
| Chloroflexi | *Anaerolinea* | 0.1 | 0 | 0 | 0 | 0 | 0 | 0 |
| Bacteroidetes | *Bacteroides* | 0 | 0 | 0 | 0 | 0 | 0 | 0.5 |
| | *Parabacteroides* | 0 | 0 | 0 | 0 | 0 | 0 | 0.4 |
| | *Proteiniphilum* | 0 | 0.1 | 0 | 0 | 0 | 0 | 0 |
| | *Chryseobacterium* | 0 | 0 | 18.3 | 0 | 0 | 26.2 | 1.7 |
| Actinobacteria | *Agromyces* | 0 | 3 | 0 | 0 | 0 | 0 | 0 |
| | *Propionibacterium* | 0 | 0 | 0 | 0 | 0.1 | 0 | 0 |
